# The Association Between Genetic Variants, Pharmacokinetics, and Infliximab Efficacy in Pediatric Patients With Crohn's Disease in China

**DOI:** 10.3389/fped.2021.744599

**Published:** 2021-12-13

**Authors:** Wenhui Hu, Yan Feng, Ziqing Ye, Zifei Tang, Lai Qian, Yuhuan Wang, Ying Huang

**Affiliations:** Department of Gastroenterology, Pediatric Inflammatory Bowel Disease Research Center, National Children's Medical Center, Children's Hospital of Fudan University, Shanghai, China

**Keywords:** Crohn's disease, infliximab, children, single nucleotide polymorphisms, pharmacokinetics

## Abstract

**Background:** Infliximab is an effective therapy for Crohn's disease (CD). Early non-invasive predictors of disease remission allow for modification of treatments. The aim of this study was to investigate the associations between genetic variants, pharmacokinetics, and infliximab efficacy in pediatric patients with CD.

**Methods:** This retrospective observational study included CD patients under infliximab therapy between August 2015 and December 2020. Information on demographics, laboratory tests, medication data, and disease activity index was collected. The trough levels of infliximab (TLI) and antibodies to infliximab (ATI) were measured at week 14, and reactive drug monitoring was performed during follow-up. Ten single-nucleotide polymorphisms involved in the NF-κB-mediated inflammatory response, pharmacokinetics, and therapeutic response to infliximab were genotyped.

**Results:** A total of 62 pediatric CD patients were enrolled. The clinical remission (CR) rate was 69.4 and 63.2% at week 14 and week 30, respectively. TLI at week 14 was significantly independently associated with CR at week 14 and mucosal healing (MH) at week 30 (*p* = 0.007 and *p* = 0.025, respectively). The optimal TLI threshold level capable of distinguishing between the CR and non-CR groups was 2.62 μg/ml (*p* < 0.001, area under the curve = 0.79, sensitivity = 69.2%, specificity = 78.9%), while that capable of distinguishing between the MH and non-MH groups was 3.34 μg/ml (*p* < 0.001, area under the curve = 0.85, sensitivity = 78.6%, specificity = 79.4%). Rs3397 in *TNFRSF1B* was associated with time to ATI production in CD patients (*p* < 0.001).

**Conclusions:** Higher TLI contributed to achieving MH. Genotyping rs3397 in *TNFRSF1B* may identify patients who are prone to generating immunogenicity to drugs.

## Introduction

Crohn's disease (CD) is a chronic inflammatory disease characterized by relapsing and remitting inflammation of the gastrointestinal tract (1). Infliximab (IFX), a chimeric human–murine monoclonal antibody against tumor necrosis factor alpha (TNF-α), has been used to induce and maintain remission in patients with CD (2). Administration of IFX rapidly improves clinical symptoms and has a durable effect for up to 54 weeks with an acceptable safety profile (3). However, a systematic review demonstrated that the annual risk of loss of response was estimated to be 13% per patient-year (4). Since IFX is the most commonly used biological agent for pediatric CD in China, optimization of IFX therapy seems to be mandatory.

Early non-invasive predictors of clinical and endoscopic remission can allow timely IFX treatment modifications in pediatric CD (5). Therapeutic drug monitoring (TDM), which is based on measurement of trough levels of IFX (TLI) and antibodies to IFX (ATI), is emerging as an important tool to optimize the efficacy of IFX in pediatric CD (6, 7). A prospective study suggested that measuring ATI and TLI might predict clinical and endoscopic remission in CD (8). Clinical interventions based on reactive TDM resulted in improved rates of disease remission (9). Higher titers of anti-drug antibodies are associated with lower drug concentrations, which may lead to immune-mediated pharmacokinetic failure (10).

Several recent studies identified genetic variants associated with abnormal therapeutic TLI and increased susceptibility to immunogenicity. The HLA-DQA1^*^05 allele was identified among European patients with luminal CD as being associated with an increased risk of developing antibodies against anti-TNF agents (11). The single-nucleotide polymorphisms (SNPs) rs5030728 (*TLR4*) and rs11465996 (*LY96*) were found to be associated with subtherapeutic TLI in pediatric CD (12). Moreover, pharmacogenetics was investigated to predict the response before anti-TNF treatment initiation. Polymorphisms in *TNFRSF1B, IL10, IL17*, and *IL6* were found to be associated with long-term response to anti-TNF therapy among pediatric CD patients, while this correlation was not found in adults, which revealed that the pharmacogenetics differs between adults and children (13). Overall, more studies are needed regarding the potential contribution of pharmacogenetics to IFX treatment for pediatric CD.

The aim of this retrospective study was to investigate the association between genetic variants, pharmacokinetics, and clinical outcomes during IFX treatment. We developed a nomogram for predicting MH at week 30 and explored the effect of SNP within a list of 10 genes on pharmacokinetics to IFX treatment in pediatric patients with CD in China.

## Methods

### Patients

This study was a retrospective single-center study conducted at the Children's Hospital of Fudan University, National Children's Medical Center, Shanghai, China between August 1, 2015, and December 1, 2020. The inclusion criteria were as follows: (1) age <18 years, (2) clinical diagnosis of CD according to the Porto criteria and Paris classification (14, 15), (3) at least 14 weeks of IFX treatment, (4) biologic-naïve patients, and (5) written informed consent. Exclusion criteria were the presence of definite germline mutation and any infections preventing the use of IFX.

This study was approved by the ethical committee at the Children's Hospital of Fudan University. Informed consent for participation and sample collection was obtained from their parents.

### Therapeutic Protocol

An induction phase with administration of IFX 5 mg/kg at weeks 0, 2, and 6 was followed by a maintenance phase, when IFX 5 mg/kg was intravenously administered every 8 weeks. From August 2015 to January 2017, IFX was used for inducing and maintaining remission in moderate-to-severe pediatric CD and in patients with a high risk of poor outcomes, including extensive disease, deep colonic ulcers, structuring, and penetrating lesions.

Increasing evidence supports that exclusive enteral nutrition (EEN) has statistically equivalent efficacy to the use of corticosteroids in inducing remission (16). EEN demonstrated better efficacy in pediatric CD patients compared to adults, as well as the added benefit of providing nutritional support during the growth phase (16, 17). Therefore, first-line EEN has been used for induction therapy in active luminal CD patients at our center since February 2017 (Supplementary Figure S1). IFX is subsequently considered for patients who (1) cannot tolerate EEN, (2) fail to achieve remission after EEN induction therapy, and (3) present high-risk factors, even in remission after induction of EEN.

Considering the potential risks of lymphoma and tuberculosis in combination with thiopurine (18), anti-TNF monotherapy is prioritized at our center. In response to the clinical evidence of disease recurrence with abnormal TLI and ATI, concomitant immunosuppressive therapy (azathioprine and methotrexate) is permitted, the IFX dose can be intensified (up to 10 mg/kg per dose), and the interval between infusions can be shortened.

### Disease Activity Assessments and Outcome Measures

Laboratory markers were monitored at each infusion, including C-reactive protein (CRP) levels, erythrocyte sedimentation rate (ESR), hematological examination, and serum biochemical indices. Clinical evaluation was performed before induction treatment and at weeks 14, 30, and 54 of IFX therapy *via* weighted Pediatric Crohn's Disease Activity Index (wPCDAI) with values indicating clinical remission (<12.5 points, CR), mild (12.5–40 points), moderate (>40–57.5 points), and severe disease (>57.5 points) (19). Colonoscopy was performed before EEN/corticosteroid induction therapy at weeks 0 and 30 of IFX therapy. A time window of endoscopic reexamination between weeks 30 and 38 was permitted. Mucosal healing (MH) was defined as the Simple Endoscopic Score for Crohn Disease (SES-CD) <3 points (20, 21).

The primary outcome was MH at week 30 of IFX therapy. Secondary outcomes included CR at weeks 14, 30, and 54 of IFX therapy and the time of ATI development.

### Measurement of TLI and ATI

TLI and ATI examinations were performed prior to infusion at week 14, and reactive drug monitoring was performed during follow-up. Peripheral blood samples were collected before IFX infusions and stored at 4°C. TLI and ATI were detected using the commercially available immunochromatographic assay, and serum TNF-α was measured using an enzyme-linked immunosorbent assay (HeRui IBD, Suzhou, China). The normal ranges for TLI, ATI, and TNF-α were >1.0 μg/ml, <30 ng/ml, and <8.1 pg/ml, respectively.

### DNA Isolation and SNP Genotyping

DNA was extracted from 250 μl of patient peripheral blood using a blood purification kit (Omega, D3392-01) according to the manufacturer's instructions. Polymorphisms were genotyped on the Agena MassARRAY® platform, conducted by OE Biotech Co., Ltd (Shanghai, China). Briefly, the initial multiple polymerase chain reaction (PCR) amplification was conducted using an Agena amplification kit. Subsequently, shrimp alkaline phosphatase treatment was performed to remove free deoxyribonucleoside triphosphates in the reaction system. Then, the single-base extension reaction was conducted, and resin purification was performed. Finally, the PCR product was genotyped by MALDI-TOF mass spectrometry and analyzed using Agena Typer 4.0 software.

A total of 10 SNPs were selected from the previous literature, namely, *TNFRSF1A* (rs4149570), *TNFRSF1B* (rs3397 and rs1061624), *TLR4* (rs5030728), *TLR2* (rs3804099), *IL6* (rs10499563), *IL17A* (rs2275913), *IL10* (rs1800872 and rs3024505), and *HLADQA1* (rs2097432), which are involved in the NF-κB-mediated inflammatory response and are associated with pharmacokinetics and the therapeutic response to IFX (11, 13, 22–24).

### Statistical Analysis

Statistical analysis was performed using SPSS 20.0 for Windows (IBM, Somers, NY). Continuous clinical and demographic variables are expressed as the median and standard deviation or as the median and interquartile range (IQR). The categorical variables are expressed as percentages. The Mann–Whitney test was applied for the comparison of two groups. Multivariate logistic regression analysis was performed on all variables with *p* < 0.05 in the univariate analysis. Receiver operating characteristic (ROC) curve analysis was performed to determine the best threshold of TLI for identifying CR or MH, as well as to calculate the specificity, sensitivity, likelihood ratio, and area under the ROC curve (AUROC). Spearman correlation analysis was performed to observe the correlation between TLI and ATI. A nomogram was formulated according to the results of the multivariate logistic regression model by applying the R package rms (25). The performance of the nomogram was internally validated in terms of calibration and discrimination capacity. The bootstrap resampling strategy was applied for the calibration plot and estimation of AUROC with 2,000 repetitions. For survival analysis, the Kaplan–Meier method was applied to estimate the survival function, and the log-rank test was used for comparison. A two-sided *p* < 0.05 significance level was used throughout.

## Results

### Clinical Characteristics and Outcomes

Among the 80 patients treated with IFX at our center, 62 CD patients met the eligibility criteria. Blood samples were obtained from 58 patients. The median duration of IFX treatment was 20.1 months (IQR: 12.6–29.87). The demographic and clinical characteristics of the enrolled patients are shown in Table 1. During the first year of IFX treatment, 9 patients (14.5%) received concomitant immunosuppressive treatment and 27 patients (43.5%) received IFX dose intensification or interval shortening.

**Table 1:** Patient's characteristics.

**Characteristic**	***N* (%)**
Gender (male/female)	39/23
Age of disease onset (years), median (IQR)	11.00 (8.00–12.41)
Age at infliximab initiation (years), median (IQR)	12.24 (10.00–14.35)
Duration of IFX therapy (months), median (IQR)	20.07 (12.60–29.87)
wPCDAI before induction therapy	37.18 ± 18.79
SES-CD before induction therapy	13.00 (7.00–19.00)
**Laboratory markers before IFX therapy**	
White blood cell (×10^9^/l)	7.05 (5.80–10.50)
Hemoglobin (g/l)	117.89 ± 16.30
Albumin (g/l)	39.19 ± 4.43
C-reactive protein (mg/l)	10.50 (8.0–24.5)
Erythrocyte sedimentation rate (mm/h)	41.50 (18.75–73.25)
**Location according to the paris classification**	
L1	8 (12.9%)
L2	19 (30.6%)
L3	34 (54.8%)
L4a	29 (46.8%)
**Behavior according to the paris classification**	
B1	53 (85.5%)
B2	6 (9.7%)
B3	3 (4.8%)
B2B3	0 (0.0%)
*P*	14 (22.6%)
**Concomitant therapy**	
5-Aminosalicylic acid	58 (93.6%)
Azathioprine	8 (12.9%)
Methotrexate	1 (1.6%)
None	4 (6.5%)

*IQR, interquartile range; wPCDAI, weighted Pediatric Crohn's Disease Activity Index; SES-CD, Simple Endoscopic Score for Crohn Disease*.

Among the 18 (29.0%) patients receiving EEN and 3 (4.8%) patients receiving corticosteroids as induction therapy, there was a significant decrease in wPCDAI and SES-CD after induction therapy (wPCDAI: 40.0, IQR: 26.25–53.75 vs. 7.5, IQR: 0.00–23.75, *p* < 0.001; SES-CD: 19.0, IQR: 13.0–20.0 vs. 5.5, IQR: 3.0–14.75, *p* = 0.002). A total of 12 (57.1%) patients were in CR before subsequent IFX therapy, and 17 (81.0%) patients achieved CR at week 14 of IFX therapy. In 41 (66.1%) patients who received anti-TNF therapy for inducing remission, there was also a significant decrease in measured values of wPCDAI, platelets, CRP, and ESR (*p* < 0.001) after induction therapy, whereas 26 (63.4%) patients were in CR after IFX induction therapy. In total, 69.4% of patients were in CR at week 14 of IFX treatment. Among the 49 patients who underwent endoscopic reexamination at week 30, MH was observed in 30.6% of patients. At week 54, 66.7% of patients (32/48) achieved CR.

### Factors Associated With Disease Remission

In order to identify risk factors that were associated with both short-term and long-term remission, we applied a two-step strategy based on a logistic regression model. In univariate logistic regression analysis, the white blood cell count, erythrocyte sedimentation rate, and wPCDAI before IFX initiation were significantly associated with CR at week 14 (Table 2). TLI was associated with CR at week 14 and MH at week 30 (both *p* = 0.002). However, the association was lost between TLI and CR at week 54 (*p* = 0.188). EEN/corticosteroid induction therapies were not statistically significant in CR and MH. Variables with a *p* < 0.05 in each univariate regression analysis were included in the multivariate analysis, as presented in Tables 2, 3.

**Table 2:** Factors associated with clinical remission at weeks 14, 30, and 54.

	**Clinical remission at week 14**				**Clinical remission at week 30**				**Clinical remission at week 54**			
	**Univariate analysis**		**Multivariate analysis**		**Univariate analysis**		**Multivariate analysis**		**Univariate analysis**		**Multivariate analysis**	
Factors	Odds ratio (95% CI)	*P*	Odds ratio (95% CI)	*P*	Odds ratio (95% CI)	*P*	Odds ratio (95% CI)	*P*	Odds ratio (95% CI)	*P*	Odds ratio (95% CI)	*P*
WBC 0W	0.861 (0.746–0.993)	**0.040**	0.941 (0.784–1.129)	0.511	0.955 (0.826–1.104)	0.536	–	–	1.046 (0.900–1.217)	0.555	–	–
ESR 0W	0.981 (0.963–0.998)	**0.028**	0.986 (0.963–1.009)	0.223	0.987 (0.971–1.004)	0.135	–	–	0.988 (0.970–1.005)	0.172	–	–
Albumin 0W	1.097 (0.965–1.247)	0.157	–	–	1.185 (1.024–1.371)	**0.023**	1.090 (0.875-1.357)	0.442	1.151 (0.981–1.349)	0.084	–	–
wPCDAI 0W	0.971 (0.944–0.998)	**0.035**	0.985 (0.947–1.025)	0.469	0.982 (0.956–1.008)	0.175	–	–	0.985 (0.958–1.014)	0.313	–	–
Platelet 14W	0.995 (0.990–1.001)	0.085	–	–	0.992 (0.986–0.999)	**0.027**	0.998 (0.988–1.007)	0.617	0.999 (0.992–1.006)	0.769	–	–
ESR 14W	–	–	–	–	0.943 (0.911–0.977)	**0.001**	1.012 (0.961–1.065)	0.654	0.960 (0.929–0.992)	**0.014**	0.955 (0.906–1.006)	0.080
Albumin 14W	–	–	–	–	1.521 (1.198–1.930)	**0.001**	1.583 (1.056–2.373)	**0.026**	1.252 (1.025–1.530)	**0.028**	1.175 (0.911–1.516)	0.215
wPCDAI 14W	–	–	–	–	0.801 (0.713–0.899)	**<0.001**	0.794 (0.656–0.961)	**0.018**	0.934 (0.869–1.005)	0.067	–	–
TLI 14W	1.900 (1.255–2.879)	**0.002**	1.851 (1.181–2.903)	**0.007**	1.385 (1.012–1.896)	**0.042**	0.864 (0.545–1.370)	0.535	1.263 (0.921–1.733)	0.148	–	–
ESR 30W	–	–	–	–	–	–	–	–	0.977 (0.955–1.000)	**0.050**	1.013 (0.975–1.054)	0.504

*WBC, white blood cell; ESR, erythrocyte sedimentation rate; wPCDAI, weighted Pediatric Crohn's Disease Activity Index; TLI, trough levels of infliximab; 95% CI, 95% confidence interval. p < 0.05, statistical significant*.

*Before induction therapy was defined as laboratory markers and disease activity score obtained before EEN/corticosteroid or infliximab induction treatment. At week 14 was defined as variables obtained at 14 weeks of infliximab therapy. Only variables with a p < 0.05 in univariate regression analysis were presented in the table and then included in multivariate analysis*.

**Table 3:** Factors associated with mucosal healing at week 30.

	**Univariate analysis**		**Multivariate analysis**	
**Factors**	**Odds ratio (95% confidence interval)**	***P***	**Odds ratio (95% confidence interval)**	***P***
**Before induction therapy**				
Albumin	1.157 (1.010–1.326)	0.035	1.230 (0.858–1.763)	0.260
ESR	0.971 (0.947–0.995)	0.018	0.987 (0.943–1.033)	0.565
SES-CD	0.872 (0.781–0.975)	0.016	0.939 (0.782–1.128)	0.501
**At week 14**				
ESR	0.917 (0.865–0.973)	0.004	1.011 (0.910–1.122)	0.845
Albumin	1.310 (1.032–1.663)	0.026	1.166 (0.708–1.923)	0.546
wPCDAI	0.873 (0.785–0.972)	0.014	0.867 (0.668–1.125)	0.281
TLI	2.333 (1.355–4.016)	**0.002**	2.759 (1.136–6.703)	**0.025**

*ESR, erythrocyte sedimentation rate; SES-CD, Simple Endoscopic Score for Crohn Disease; wPCDAI, Weighted Pediatric Crohn's Disease Activity Index; TLI, trough levels of infliximab. p < 0.05, statistical significant*.

*Before induction therapy was defined as laboratory markers and disease activity score obtained before EEN/corticosteroid or infliximab induction treatment. At week 14 was defined as variables obtained at 14 weeks of infliximab therapy. Only variables with a p < 0.05 in univariate regression analysis were presented in the table and then included in multivariate analysis*.

Multivariate logistic regression analysis was then performed to assess the independent factors associated with clinical response and MH. Higher TLI at week 14 independently predicted a higher probability of MH at week 30 (Table 3). Consistent with the findings from univariate analyses, TLI was confirmed to be an independent predictor for short-term treatment response (Table 2). The median TLI at week 14 was significantly higher in patients who achieved CR at week 14 compared with those who did not (3.50 vs. 0.47 μg/ml, *p* < 0.001), as well as in patients with MH compared with those without MH (4.35 vs. 1.43 μg/ml, *p* < 0.001).

**Figure 1:**
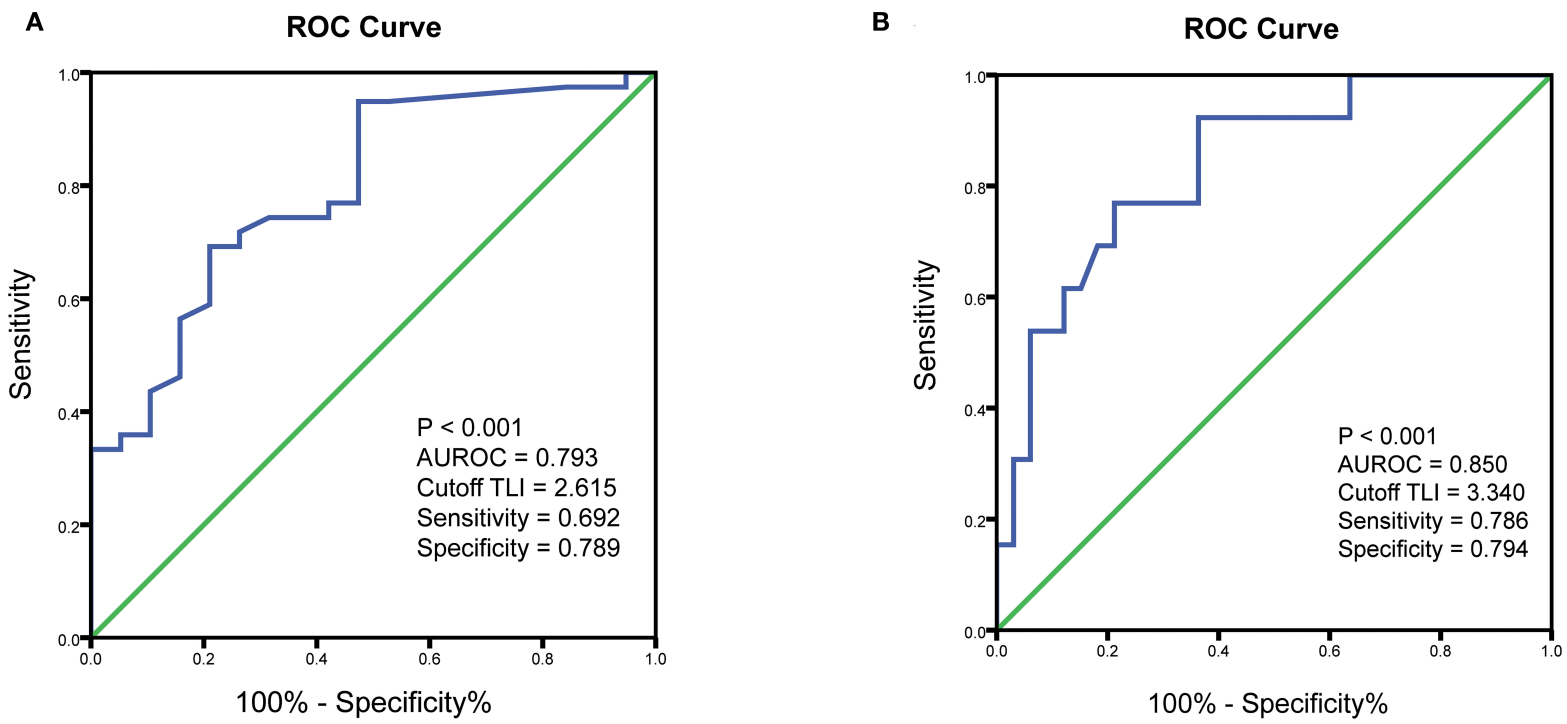
ROC curves showing the association of TLI with clinical outcomes. **(A)** ROC curve to identify the threshold value of TLI associated with clinical remission. **(B)** ROC curve to identify the optimal cutoff for TLI associated with endoscopic remission. ROC, receiver operating characteristic; TLI, trough levels of infliximab; AUROC, area under the ROC curve.

ROC curves were constructed to determine the optimal cutoff value of TLI at week 14 for predicting CR and MH in pediatric CD patients treated with IFX. The optimal TLI threshold level capable of distinguishing between the CR and non-CR groups at week 14 was 2.62 μg/ml with an AUROC of 0.79 (*p* < 0.001, sensitivity = 69.2%, specificity = 78.9%). Furthermore, the threshold value of TLI at week 14 capable of differentiating patients with or without MH at week 30 was 3.34 μg/ml with an AUROC of 0.85 (*p* < 0.001, sensitivity = 78.6%, specificity = 79.4%) (Figure 1).

No significant differences in CR or MH rate in the first year were found on the basis of SNP genotype.

### Prognostic Nomogram for MH at Week 30

**Figure 2:**
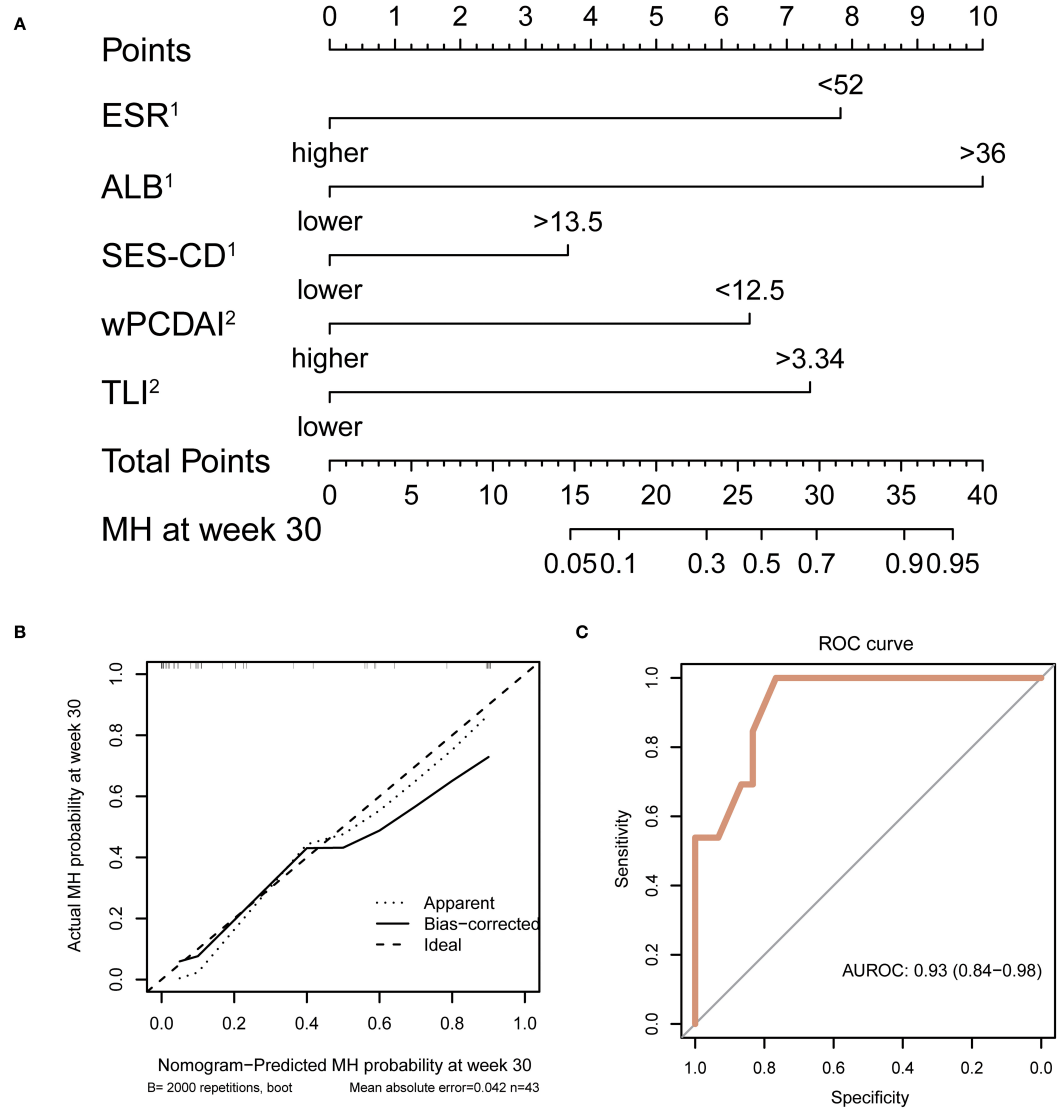
Development, calibration, and performance of nomogram. **(A)** Week 30 MH nomogram. Each variable is assigned a score on the “points” axis. The sum point corresponds to the estimated probability of MH at week 30. ESR, ALB, and SES-CD were evaluated before induction treatment. wPCDAI and TLI were determined at week 14. **(B)** Calibration plot for internal validation of the nomogram. Curves represent observed vs. predicted probabilities. **(C)** ROC curve indicating the discrimination capacity of the nomogram. AUC and its 95% confidence interval were estimated by bootstrap resampling with 2,000 repetitions. MH, mucosal healing; ESR, erythrocyte sedimentation rate; ALB, albumin; SES-CD, Simple Endoscopic Score for Crohn Disease; wPCDAI, weighted Pediatric Crohn's Disease Activity Index; AUC, area under the curve.

A nomogram was then constructed to facilitate the prediction of MH at week 30. The variable selection was in accordance with the results of the unadjusted logistic regression model of MH at week 30. Factors including ESR, albumin, and SES-CD before induction treatment, as well as wPCDAI and TLI at week 14, were used as dichotomous variables with the cut-off values determined by Youden index. The final nomogram is depicted in Figure 2A. The calibration capacity of the multivariate model was visually inspected using the calibration chart (Figure 2B) showing great agreement between actual and predicted probabilities. On the other hand, the discrimination capacity of the model was assessed by ROC analysis with an AUROC reaching 0.93 (95% confidence interval: 0.84–0.98) (Figure 2C). Of note, we observed that the predicted probability closely approximated the actual observation in the lower range of probability from 0 to 0.4, indicating that our model may potentially help identify patients failing to achieve MH at the earliest time point of week 14.

### SNPs Associated With Pharmacokinetics to IFX in CD

The ATI level was below the detection limit (<4 ng/ml) in 74.2% (46/62) of patients at week 14. Although ATI at week 14 was not associated with disease remission at weeks 14, 30, and 54, ATI levels were inversely correlated with TLI during the maintenance treatment (Pearson test *p* = 0.002, ρ = −0.392). As the IFX treatment progressed, 16 of 62 patients (25.8%) showed positively increased antibody levels (defined as ATI >30 ng/ml). The median time from inception to ATI detection in our population was 13.48 months (IQR: 8.57–23.02).

**Figure 3:**
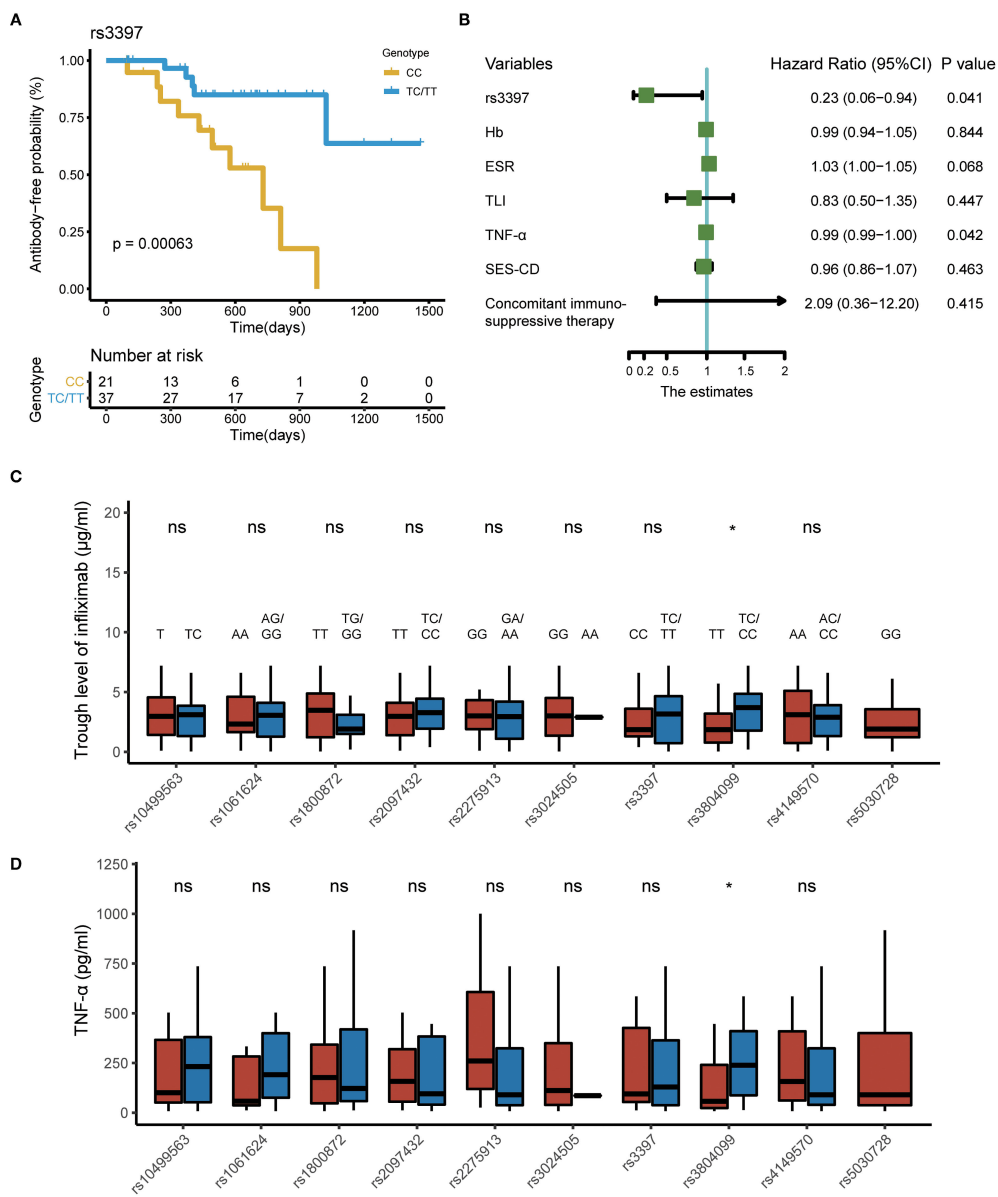
SNPs associated with pharmacokinetics of IFX in CD. **(A)** Kaplan–Meier curve depicting the difference in antibody-free probability between patients with the CC genotype and those with the TC/TT genotype. **(B)** Forest plot showing the results of multivariate Cox regression model. **(C)** Polymorphisms associated with TLI at week 14. **(D)** Polymorphisms associated with serum TNF-α level at week 14. SNPs, single-nucleotide polymorphisms; IFX, infliximab; CD, Crohn's disease; TNF-α, tumor necrosis factor alpha.

In the time-to-event analysis, one SNP (rs3397) in *TNFRSF1B* was significantly associated with the time of ATI production. However, associations between the nine other SNPs and ATI development were not statistically significant. *TNFRSF1B* CC genotype (rs3397) was predictive of significantly earlier ATI occurrence compared to the TC or TT genotype with the *p* < 0.001 (Figure 3A). We noticed that the probability of remaining antibody-free 3 years after the start of IFX therapy was more than 50% in patients with a TC or TT genotype. After adjusting for variables including hemoglobin, erythrocyte sedimentation rate before IFX treatment, TLI at week 14, serum TNF-α level at week 14, SES-CD score before IFX treatment, and concomitant immunosuppressive therapy, *TNFRSF1B* CC genotype remained a significant risk factor for the generation of ATI (Figure 3B).

Moreover, compared with patients carrying the rs3804099 TC and CC genotype, those carrying the rs3804099 TT genotype had a lower median TLI (3.7 vs. 2.1 μg/ml, *p* = 0.039) and serum TNF-α level (239.0 vs. 71.8 pg/ml, *p* = 0.034) at week 14. The remaining SNPs were not statistically significant in the comparison of TLI and TNF-α level at week 14 (Figures 3C,D).

## Discussion

In the present study, 62 patients with CD treated with IFX were evaluated, and factors associated with CR and MH were identified. Notably, we identified one SNP (rs3397) as an independent risk factor for the presence of serum ATI. To our knowledge, this is the first study to investigate the role of pharmacogenetics in ATI development among pediatric patients with CD in China.

The association between TLI and clinical or endoscopic outcomes was observed at weeks 14 and 30. Singh et al. found that TLI and CRP at week 14 were significantly associated with persistent efficacy within 1 year (26). Ungar et al. found that TLI >2.2 μg/ml at week 6 could predict sustained response for 1 year of IFX treatment (7). However, a relationship between TLI and long-term clinical response was not observed in this study, which was possibly due to the dose adjustment in some patients during the maintenance treatment and the small sample size.

Some studies showed that higher TLIs were associated with better clinical outcomes (27). A prospective observational study revealed that the optimal cutoff for TLI associated with CR and CRP normalization was 2.1 μg/ml, while that for TLI associated with CR with normal CRP and fecal calprotectin (<50 μg/g) was 4.9 μg/ml in adults with inflammatory bowel disease (IBD) (28). Another observational study reported that TLI >3.11 μg/ml at week 14 predicted sustained CR (week 54) in pediatric IBD (29). For predicting MH, Chaparro et al. demonstrated that the best cutoff point of TLI was 3.4 μg/ml with an AUROC of 0.63 in adult patients (30). In this study, the optimal cutoff value of TLI for identifying patients in MH (3.34 μg/ml) was consistent with some previously reported data for adults (28, 30). IFX pharmacokinetic properties were comparable between pediatric and adult CD patients (31). On the other hand, the cutoff levels in our study were also lower than some other studies. Kang et al. considered that the cutoff value of TLI for achieving MH was ≥5 μg/ml with a specificity of 80% in children (32). However, the definition of MH in that study was an SES-CD of 0 point. A more stringent expected level of clinical outcome was associated with a higher optimal threshold (28). Another retrospective study proposed that a therapeutic level of 6–10 μg/ml for IFX was required for MH, but the trough levels of blood samples were not detected (33). Moreover, the individual variations in pharmacokinetics of IFX, such as gender, ethnicity, body mass index, development of immunogenicity, and disease severity, influenced the IFX pharmacokinetics (34). Significantly, there might be substantial discrepancies between different commercial enzyme-linked immunosorbent assay kits for monitoring TLI (35). Therefore, it is recommended to use the same assay during follow-up and cautiously compare results issued by different kits.

Furthermore, we found that the ATI detection rate was quite low at week 14, which might have been due to the early testing time point. As the treatment progressed, the number of patients with positive ATI levels increased, and the median time to antibody detection was 13.48 months. Our result is consistent with a retrospective study that revealed a time to antibody detection for IFX of 14.83 months (36). In contrast to one previous study, which demonstrated that ATI in the early treatment course could predict long-term outcomes (37), no association between ATI and clinical outcomes was observed in the present study. Nevertheless, TLI was inversely correlated with ATI levels during the maintenance treatment, and factors affecting ATI occurrence were assessed.

During the evaluation of SNPs, one SNP (rs3397) in the *TNFRSF1B* gene was found to be significantly associated with time to ATI production. Interestingly, rs3397 was previously found to be associated with long-term response to anti-TNF treatment in pediatric CD patients (13). *TNFRSF1B* (TNF receptor superfamily 1B) is a single transmembrane glycoprotein that can induce cell survival or cell apoptosis (38). A recent study demonstrated that the C allele of rs3397 was associated with subtherapeutic trough serum levels of adalimumab (<5 μg/ml) (12). No data about this SNP have been reported in terms of ATI production. Although rs3397 was not found to be related to TLI in our study, the multivariate Cox regression analysis confirmed that the C allele for SNP rs3397 in *TNFRSF1B* was associated with a higher risk of earlier ATI production. The poorer outcome for patients diagnosed with CD with the CC genotype in rs3397 than in those with CT or TT suggests that genotyping this SNP could help to identify patients who are prone to immunogenicity to drugs. Bank et al. (23) reported that the TC or CC genotype of rs3804099, another polymorphism in *TLR2* regulating the activation of the NF-κB pathway, was associated with a beneficial response among CD patients. However, our observation demonstrated that patients carrying the rs3804099 TC and CC genotype expressed higher TLI and serum TNF-α levels at week 14. Since an early increase in soluble TNF-α during IFX therapy might reflect a better long-term clinical outcome (39), our finding provides more evidence for the potential clinical application of rs3804099. Notably, rs5030728 in *TLR4* was identified to be associated with subtherapeutic TLI in Spanish pediatric IBD (12), but all patients detected for rs5030728 were G allele carriers in our cohort, which might indicate a difference in pharmacogenetics among various populations. More studies are required to further confirm the role of this SNP as a biomarker for IFX treatment in pediatric CD.

The limitations of this study were its retrospective design, the relatively small number of enrolled patients, and missing data. Considering that the incidence of pediatric IBD is lower in China than that in Western countries (40) and that this center is one of the largest pediatric IBD centers in China, the enrolled population is considered representative. Moreover, a longer follow-up time may have resulted in a higher ATI detection rate compared with this present study. Despite these limitations, the identification of the role of TLI in clinical efficacy and SNP in ATI development provides more evidence that TDM or genotyping may optimize IFX treatment in pediatric CD patients in China.

In conclusion, this study indicates an association between TLI and short-term treatment outcomes in pediatric CD patients. The identification of the cutoff value for TLI contributing to MH in pediatric CD patients may guide adjustments of drug dosing in the treat-to-target approach. The C allele for SNP rs3397 in *TNFRSF1B* was found to be associated with a higher risk of earlier ATI production.

## Data Availability Statement

The original contributions presented in the study are included in the article/Supplementary Materials, further inquiries can be directed to the corresponding author/s.

## Ethics Statement

This study was approved by the ethical committee at the Children's Hospital of Fudan University. Informed consents for participation and samples collection were obtained from their parents.

## Author Contributions

WH performed the experiments, analyzed the data, and drafted the manuscript. ZY and YH revised the manuscript. YF, ZT, YW, and LQ were responsible for the associated data collection. All authors contributed to the article and approved the submitted version.

## Funding

This study was supported by the National Children's Medical Center, Haiju International Joint Lab Fund (EK1125180105).

## Conflict of Interest

The authors declare that the research was conducted in the absence of any commercial or financial relationships that could be construed as a potential conflict of interest.

## Publisher's Note

All claims expressed in this article are solely those of the authors and do not necessarily represent those of their affiliated organizations, or those of the publisher, the editors and the reviewers. Any product that may be evaluated in this article, or claim that may be made by its manufacturer, is not guaranteed or endorsed by the publisher.

## Supplementary Material

Supplementary Figure S1 Illustration of infliximab therapeutic protocol for pediatric CD patients at our center. IFX, infliximab; EEN, exclusive enteral nutrition; TDM, Therapeutic drug monitoring.
