# Supplementary figures and images for: The Association Between Genetic Variants, Pharmacokinetics, and Infliximab Efficacy in Pediatric Patients With Crohn's Disease in China
Source: Front Pediatr. 2021 Dec 13;9:744599. doi: 10.3389/fped.2021.744599 (PMC8711600; doi:10.3389/fped.2021.744599)

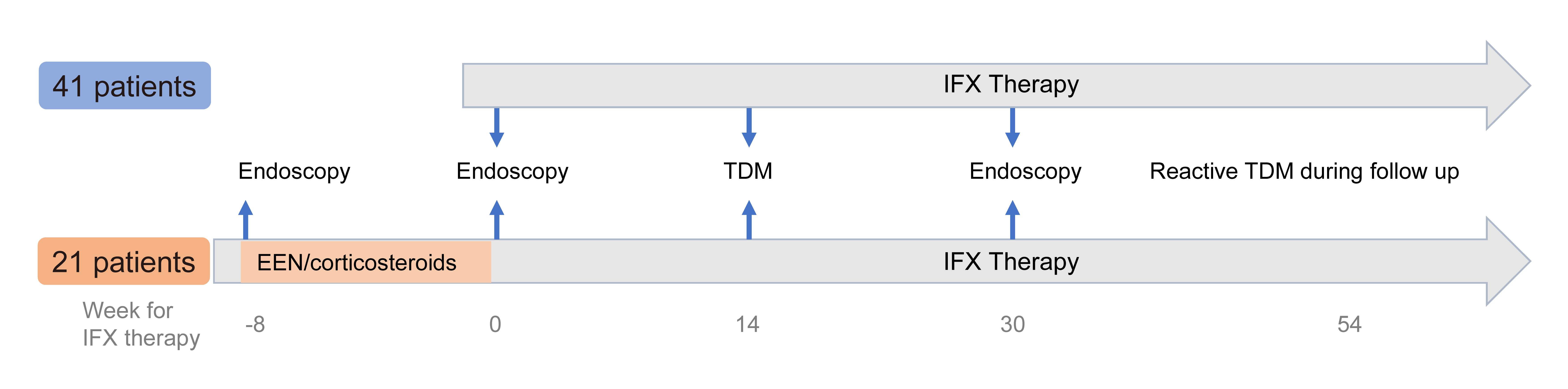

Supplement: Supplementary Figure S1 — Illustration of infliximab therapeutic protocol for pediatric CD patients at our center. IFX, infliximab; EEN, exclusive enteral nutrition; TDM, Therapeutic drug monitoring. [file Image_1.tif]
